# Staurosporine Drives Non‐Canonical Melanocyte Maturation by Coupling β‐Catenin Signaling to Actin‐Dependent Dendrite Remodeling

**DOI:** 10.1111/pcmr.70116

**Published:** 2026-08-13

**Authors:** Kangning Xu, Lingli Yang, Sylvia Lai, Fei Yang, Yasutaka Kuroda, Daisuke Tsuruta, Ichiro Katayama

**Affiliations:** ^1^ Department of Pigmentation Research and Therapeutics, Graduate School of Medicine Osaka Metropolitan University Osaka Japan; ^2^ Department of Dermatology, Graduate School of Medicine Osaka Metropolitan University Osaka Japan; ^3^ Skin Beauty 3rd Research Laboratories, Research and Development Kao Corporation Odawara Japan

**Keywords:** dendricity, melanocyte, pigmentation, staurosporine

## Abstract

Skin pigmentation relies on the coordinated regulation of melanin production and dendritic morphology to ensure effective pigment distribution. While staurosporine is widely used as a pro‐apoptotic agent in malignant cells, its effects on normal human melanocytes remain poorly understood. Here, we investigated the effects of staurosporine on melanocyte biology and identified it as a potent inducer of non‐canonical melanocyte maturation at sub‐cytotoxic concentrations without stimulating melanocyte proliferation. In primary human melanocytes, staurosporine enhanced melanogenesis and promoted dendrite formation, elongation, and branching, resulting in acquisition of a mature melanocyte phenotype distinct from its apoptotic effects in melanoma cells. These phenotypic changes were accompanied by activation of β‐catenin signaling together with coordinated remodeling of Rho family protein expression and the actin cytoskeleton. Topical application of staurosporine increased physiological pigmentation in guinea pig skin without detectable inflammation or melanocyte proliferation. Furthermore, staurosporine accelerated repigmentation in a rhododendrol‐induced leukoderma model by promoting functional maturation of epidermal melanocytes and enhancing nuclear localization of β‐catenin. Collectively, our findings identify staurosporine as a non‐canonical regulator of melanocyte maturation and demonstrate that functional maturation, rather than proliferative expansion, is sufficient to enhance pigmentation under both physiological and depigmented conditions.

## Summary

- Staurosporine promotes non‐canonical maturation of human melanocytes at sub‐cytotoxic concentrations.
- Treatment enhances both melanogenesis and dendritic remodeling, supporting functional pigmentation.
- Staurosporine increases baseline skin pigmentation in vivo without inducing inflammation.
- Repigmentation is accelerated in a rhododendrol‐induced leukoderma model through restoration of mature melanocyte populations.
- These findings highlight coordinated regulation of pigment production and dendritic morphology as a potential strategy to promote pigmentation in acquired hypopigmentary conditions.

## Graphical abstract

Staurosporine drives non‐canonical melanocyte maturation through β‐catenin‐dependent actin remodeling. Functional maturation enhances dendrite architecture and melanogenic differentiation without inducing melanocyte proliferation, providing a biological framework for restoring functional melanocytes in acquired hypopigmentary disorders.

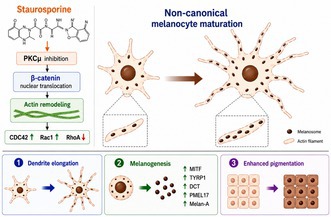

## 1 Introduction

Melanocytes are specialized neural crest‐derived cells located in the basal layer of the epidermis, where they synthesize melanin within melanosomes and transfer these organelles to surrounding keratinocytes. This process relies on an elaborate dendritic network that enables efficient melanosome transport and distribution, thereby determining skin pigmentation and contributing to photoprotection against ultraviolet radiation (Lin and Fisher 2007; Westerhof 2006). Accordingly, melanocyte dendricity is not merely a morphological characteristic but a critical functional determinant of the epidermal melanin unit (Fitzpatrick and Breathnach 1963; Lin and Fisher 2007). Disruption of dendritic architecture frequently precedes visible pigment loss and is a shared feature of multiple hypopigmentary conditions, including vitiligo, post‐inflammatory hypopigmentation, and chemically induced leukoderma (Lin and Fisher 2007). Despite its central importance, regulatory mechanisms that coordinate melanocyte dendritic remodeling with pigmentation remain incompletely understood.

Staurosporine is a microbial alkaloid originally characterized as a potent, broad‐spectrum kinase inhibitor (Omura et al. 1995; Tamaoki et al. 1986) and has long been used as a canonical inducer of apoptosis in melanoma and other cancer cells (Bertrand et al. 1994; Gescher 1998; Zhang et al. 2004). At sub‐cytotoxic concentrations, however, staurosporine has been reported to exert diverse biological effects in non‐malignant cells, particularly in neuronal lineages, where it promotes neurite outgrowth (Kohno et al. 2015; Maeda et al. 1992; Sano et al. 1994; Thompson and Levin 2010), morphological differentiation (Borge et al. 1997; Rasouly et al. 1996; Thompson and Levin 2010), and cytoskeletal reorganization (Borge et al. 1997; Hoben and Athanasiou 2008; Kim et al. 2012; Kohno et al. 2015; Sano et al. 1994). These observations suggest that staurosporine can influence cell shape and differentiation programs in a context‐dependent manner. Whether similar non‐apoptotic, morphogenetic effects occur in normal human melanocytes has not been systematically examined.

Here, we report that staurosporine elicits fundamentally distinct responses in normal melanocytes compared with melanoma cells. While melanoma cells undergo apoptosis in response to staurosporine, primary human melanocytes exhibit marked resistance at comparable concentrations. Instead, staurosporine robustly enhances melanocyte dendrite remodeling, pigmentation, and functional maturation. These effects are associated with coordinated regulation of cytoskeletal dynamics and melanogenic activity, indicating activation of a non‐canonical maturation program. We further demonstrate the physiological relevance of this response in vivo, as topical staurosporine application increases pigmentation in guinea pig skin and accelerates repigmentation in a rhododendrol‐induced leukoderma model. Together, our findings identify staurosporine as a powerful modulator of melanocyte morphology and pigmentation and reveal an intrinsic pathway linking dendritic remodeling to pigment production.

## 2 Materials and Methods

### 2.1 Reagents

Staurosporine (purity > 99%) was obtained from Cell Signaling Technology Inc. (CST; Product No. 9953; Danvers, MA, USA). For in vitro experiments, staurosporine (STS) was dissolved in DMSO. For in vivo experiments, it was formulated in a 50% ethanol/50% sesame oil (*v/v*) mixture. For all in vitro treatments, DMSO concentrations were adjusted to a final concentration of 0.1% (*v/v*) in the growth medium. RD (4‐(4‐hydroxyphenyl)‐2‐butanol; trade name Rhododenol) was generously provided by Kanebo Cosmetics Inc. (Tokyo, Japan). A 20% (*w/v*) rhododendrol stock solution was prepared in a 50% ethanol/50% sesame oil (*v/v*) mixture.

### 2.2 Cell Lines and Cell Culture

HEMn‐MPs (normal human primary epidermal melanocytes derived from moderately pigmented neonatal foreskin) were obtained from Invitrogen (Carlsbad, CA, USA) and cultured in medium 254 (M‐254‐500; Thermo Fisher Scientific, Waltham, MA, USA) supplemented with 1% (*v/v*) human melanocyte growth supplement. Human melanoma cell lines G‐361 and MEWO were obtained from the Japanese Collection of Research Bioresources (JCRB, Osaka, Japan) and maintained in high‐glucose Dulbecco's modified Eagle medium (DMEM) supplemented with 10% fetal bovine serum and 1% penicillin–streptomycin (Thermo Fisher Scientific). All cultures were at 37°C in a humidified atmosphere containing 5% CO_2_.

### 2.3 RNA Interference

For siRNA‐mediated knockdown of β‐catenin, GSK3β, CDC42, and Rac1, HEMn‐MPs were transfected with 30 nM predesigned Silencer Select siRNAs (si‐β‐catenin: s436; si‐GSK3β: s6240; si‐CDC42: VHS40396; si‐Rac1: s11712; Thermo Fisher Scientific) using Lipofectamine RNAiMAX (Invitrogen), in accordance with the manufacturer's instructions.

### 2.4 Cell Viability Assay

HEMn‐MPs and human melanoma cells (G‐361 and MEWO) were seeded in 96‐well flat‐bottom tissue culture plates at a density of 2.5 × 10^4^ cells/well. After the indicated treatments, cells were washed three times with cold phosphate‐buffered saline (PBS). Cell viability was assessed using the Cell Count Reagent SF colorimetric assay (Nacalai Tesque, Kyoto, Japan) according to the manufacturer's instructions. Briefly, 10 μL of Cell Count Reagent SF was added to each well, followed by incubation at 37°C for 2 h. Absorbance was measured at 450 nm using a microplate reader (Model 550; Bio‐Rad Laboratories, Hercules, CA, USA). Cell viability was expressed as the percentage of the DMSO‐treated control, calculated as (T/C) × 100, where T and C represent the mean OD_450_ values of the treated and control groups, respectively.

### 2.5 Cell Treatment

HEMn‐MPs and human melanoma cells (G‐361 and MEWO) were seeded and allowed to adhere overnight before treatment with DMSO or the indicated concentrations of STS for specified durations. Following treatment, cells were harvested or processed for the subsequent analyses described below.

### 2.6 Time‐Lapse Live Imaging and Cell Tracking Analysis

HEMn‐MPs were cultured in ibiTreat dishes (ibidi, Martinsried, Germany) and maintained at 37°C in a humidified atmosphere containing 5% CO_2_. Time‐lapse images were acquired every 15 min for 24 h using a Biozero BZ‐8100 fluorescence microscope (Keyence Corporation, Osaka, Japan). Melanocytes were identified based on their characteristic dendritic morphology. Individual cell movements were analyzed using the Manual Tracking plugin in ImageJ software (National Institutes of Health, Bethesda, MD, USA). Briefly, individual melanocytes were manually tracked from the first frame until they exited the field of view or the recording ended. Tracking data were exported to Microsoft Excel (Microsoft Corporation, Redmond, WA, USA) for further analysis. Migration velocity, total path length, and displacement were calculated from the tracking data, and representative cell trajectories were generated to visualize migration behavior.

### 2.7 Quantification of Dendrite Morphology

Bright‐field images of HEMn‐MPs were acquired after treatment with DMSO or staurosporine under the indicated conditions. Individual melanocytes were analyzed using ImageJ software (National Institutes of Health, Bethesda, MD, USA). Dendrite number was determined by counting the number of primary dendritic processes extending from each melanocyte. Dendrite length was defined as the mean distance from the dendrite tip to the center of the nucleus. Dendrite branching was quantified as the number of branch points per dendrite. At least 100 melanocytes were analyzed for each experimental condition.

### 2.8 DOPA Reaction Assay

HEMn‐MPs were treated with DMSO or staurosporine under the indicated conditions and washed twice with phosphate‐buffered saline (PBS). Cells were then incubated with 0.1% L‐3,4‐dihydroxyphenylalanine (L‐DOPA; Sigma‐Aldrich, St. Louis, MO, USA) prepared in phosphate buffer (pH 6.8) at 37°C for 1 h. After incubation, cells were washed with PBS and observed using a Biozero BZ‐8100 fluorescence microscope (Keyence Corporation, Osaka, Japan).

### 2.9 Phospho‐Kinase Array

Phosphorylated kinases were profiled using the Proteome Profiler Human Phospho‐Kinase Array Kit (R&D Systems, Minneapolis, MN, USA). Procedures were performed following the manufacturer's protocol with 300 μg of protein lysate per array.

### 2.10 Immunoblot Analysis

For protein preparation, cell pellets were extracted as previously described (Yang et al. 2018), and 5 μg of protein were utilized for immunoblot analysis. Primary antibodies were applied at the following dilutions: anti‐PARP (CST) 1:1000; anti‐cleaved PARP (CST) 1:1000; anti‐caspase‐3 (CST) 1:1000; anti‐cleaved caspase‐3 (CST) 1:1000; anti‐MITF (Santa Cruz Biotechnology, Dallas, TX, USA) 1:500; anti‐TYRP1 (Santa Cruz Biotechnology) 1:500; anti‐Melan‐A (Abcam, Cambridge, UK) 1:500; anti‐PMEL17 (Santa Cruz Biotechnology) 1:500; anti‐EDNRB (Abcam) 1:1000; anti‐phosphorylated PKCμ (p‐PKCμ, Ser744/748; CST) 1:1000; anti‐p‐PKCμ (Ser916; CST) 1:1000; anti‐PKCμ (CST) 1:1000; anti‐phosphorylated GSK3α/β (p‐GSK3α/β, Ser21/9; CST) 1:1000; anti‐GSK3α/β (CST) 1:1000; anti‐phosphorylated β‐catenin (p‐β‐catenin, Ser675; CST) 1:1000; anti‐β‐catenin (CST) 1:1000; anti‐CDC42 (CST) 1:500; anti‐Rac1/2/3 (CST) 1:500; anti‐RhoA (CST) 1:500; anti‐RhoB (CST) 1:1000; anti‐RhoC (CST) 1:1000; and anti–glyceraldehyde‐3‐phosphate dehydrogenase (GAPDH; CST) 1:1000. GAPDH served as a loading control.

### 2.11 Immunofluorescence Staining of Cells

HEMn‐MPs and human melanoma cells (G‐361 and MEWO) were seeded in six‐well plates and treated with DMSO or staurosporine at the indicated concentrations and time points. After treatment, cells were washed thoroughly with ice‐cold phosphate‐buffered saline and fixed with 4% paraformaldehyde for 5 min. Fixed cells were stained with anti‐cleaved caspase‐3 (CST) or anti‐active β‐catenin (CST) at a 1:100 dilution. Nuclei were counterstained with Hoechst 33342 (Invitrogen) at a 1:500 dilution. Images were acquired using a Biozero 8100 confocal microscope (Keyence Corporation).

### 2.12 Animals and In Vivo Experimental Procedures

Six male Kwl:JY‐4 black guinea pigs (6 weeks of age) were obtained from Tokyo Laboratory Animals Science (Tokyo, Japan) and maintained under specific pathogen‐free conditions. After dorsal hair removal with an electric shaver, three or four discrete 1 × 1 cm skin areas were designated on each animal and assigned to different experimental conditions. Each experimental group included three guinea pigs. In the normally pigmented skin model, designated areas received daily topical application of vehicle or staurosporine at concentrations of 250, 500, or 1000 μM for 14 consecutive days. In the RD‐treated skin model, leukoderma was induced by daily topical application of 30% (*w/v*) RD for 30 days. After cessation of RD treatment, depigmented areas were treated daily with vehicle or staurosporine (1000 μM) for an additional 14 days. Skin samples were collected 24 h after the final topical treatment. All efforts were made to minimize animal suffering. All procedures were conducted in accordance with the Guiding Principles for the Care and Use of Laboratory Animals and were approved by the Committee for Animal Experiments at Osaka Metropolitan University (Permit No. 18042).

### 2.13 Measurement of Skin Color

Skin color in treated areas was measured using a portable reflectance spectrophotometer (CM‐26d; Konica Minolta, Tokyo, Japan). Color values were expressed as L* values, representing skin lightness on a scale from 0 (black) to 100 (white).

### 2.14 Fluorescent Immunohistochemical Staining of Guinea Pig Skin Tissues

Dorsal skin tissues were fixed in 10% formaldehyde and embedded in paraffin. Paraffin sections (5 μm) were prepared for fluorescent immunohistochemical staining. Sections were deparaffinized, rehydrated, and incubated overnight at 4°C with primary antibodies against TYRP1 (1:200; Sigma‐Aldrich) and β‐catenin (1:200; CST). After they had been washed, sections were incubated with species‐appropriate Alexa Fluor‐conjugated secondary antibodies (anti‐rabbit IgG Alexa Fluor 488 and anti‐mouse IgG Alexa Fluor 555; Invitrogen). Nuclei were counterstained with Hoechst 33342 (1:500; Invitrogen). Images were acquired using a fluorescence microscope or a Biozero 8100 confocal microscope (Keyence Corporation).

### 2.15 Hematoxylin and Eosin Staining of Guinea Pig Skin Tissues

Dorsal skin tissues were fixed in 10% formaldehyde, embedded in paraffin, and sectioned at 5 μm. Sections were deparaffinized in xylene, rehydrated through graded ethanol solutions, and stained with hematoxylin and eosin according to standard protocols. After they had been stained, sections were rinsed with water, dehydrated through graded ethanol, and mounted for imaging.

### 2.16 Statistical Analyses

All experiments were performed at least three times. Data are presented as mean ± standard deviation (SD). Statistical analyses were performed using Microsoft Excel (Microsoft Corporation, Redmond, WA, USA). Comparisons between two groups were analyzed using an unpaired two‐tailed Student's *t*‐test. Comparisons among multiple groups were analyzed by one‐way analysis of variance (ANOVA) followed by Bonferroni's multiple comparisons test. A *p* value < 0.05 was considered statistically significant.

## 3 Results

### 3.1 Staurosporine Induces Apoptosis in Melanoma Cells but Not Normal Human Melanocytes

**FIGURE 1:**
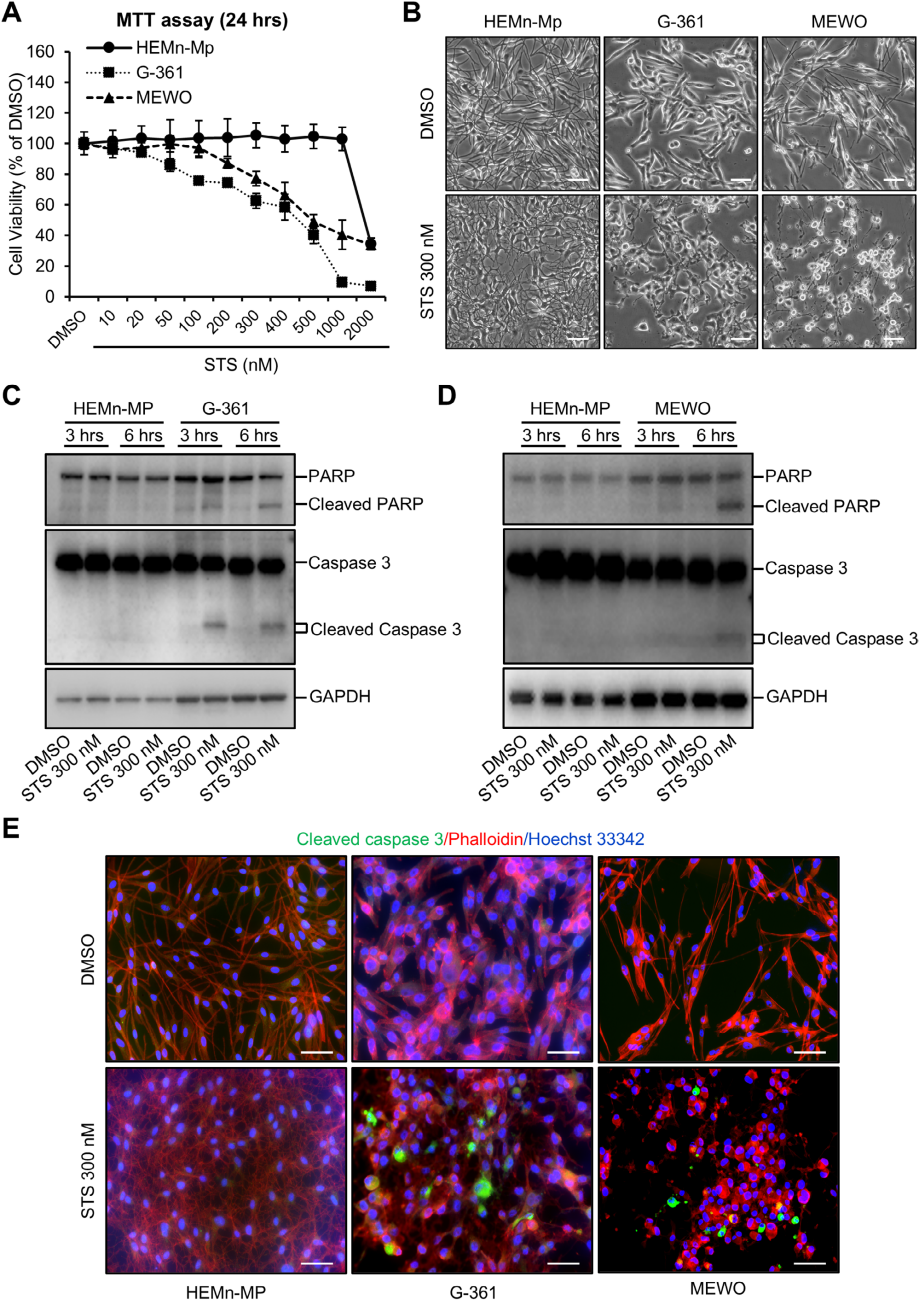
Differential effects of staurosporine on cell viability and apoptosis in normal melanocytes and melanoma cells. (A) Cell viability of normal human epidermal melanocytes (HEMn‐MP) and melanoma cell lines (G‐361 and MEWO) following treatment with increasing concentrations of staurosporine (STS) for 24 h. Cell viability is expressed as a percentage of the DMSO‐treated control. Data are presented as the mean ± SD of three independent experiments. (B) Representative bright‐field images of HEMn‐MP, G‐361, and MEWO cells treated with DMSO or 300 nM STS for 24 h. Scale bars, 200 μm. (C) Immunoblot analysis of PARP, cleaved PARP, caspase‐3, and cleaved caspase‐3 in HEMn‐MP and G‐361 cells treated with DMSO or 300 nM STS for 3 or 6 h. GAPDH served as a loading control. (D) Immunoblot analysis of PARP, cleaved PARP, caspase‐3, and cleaved caspase‐3 in HEMn‐MP and MEWO cells treated with DMSO or 300 nM STS for 3 or 6 h. GAPDH served as a loading control. (E) Representative immunofluorescence images of cleaved caspase‐3 (green), F‐Actin stained with phalloidin (red), and nuclei stained with Hoechst 33342 (blue) in HEMn‐MP, G‐361, and MEWO cells treated with DMSO or 300 nM STS. Scale bars, 50 μm.

To determine whether staurosporine exerts distinct effects in normal melanocytes and melanoma cells, we compared its impact on cell viability and apoptosis in primary normal human epidermal melanocytes (HEMn‐MP) and two human melanoma cell lines, G‐361 and MEWO. Cell viability assays revealed a pronounced cell type‐specific response to staurosporine treatment (Figure 1A). In both G‐361 and MEWO melanoma cells, staurosporine caused a pronounced, dose‐dependent reduction in cell viability, whereas HEMn‐MP melanocytes maintained high viability across the same concentration range. Notably, melanocytes exhibited minimal viability loss even at concentrations that were strongly cytotoxic to melanoma cells, indicating robust resistance to staurosporine‐induced cell death. Consistent with these findings, phase‐contrast microscopy demonstrated marked morphological changes in both melanoma cell lines after staurosporine treatment, including cell shrinkage and loss of adherence, whereas HEMn‐MP melanocytes showed no apparent cytotoxic changes or cell detachment (Figure 1B). These observations suggested that staurosporine selectively triggers cytotoxic responses in melanoma cells but not normal melanocytes. We next examined whether the differential effects of staurosporine on cell viability were associated with apoptosis. Immunoblot analysis demonstrated robust PARP cleavage and caspase‐3 activation in both G‐361 and MEWO cells after staurosporine treatment, whereas no detectable cleavage of PARP or caspase‐3 was observed in HEMn‐MP melanocytes (Figure 1C,D). These results indicate that staurosporine induces classical caspase‐dependent apoptosis in melanoma cells but fails to activate apoptotic signaling in normal melanocytes. Immunofluorescence staining further confirmed these findings. Cleaved caspase‐3‐positive cells were readily detected in both G‐361 and MEWO melanoma cells following staurosporine treatment, whereas HEMn‐MP melanocytes remained largely negative for cleaved caspase‐3 despite exposure to the same concentration of staurosporine (Figure 1E). To further exclude the possibility that staurosporine affects melanocyte proliferation, Ki‐67 expression was examined by both immunofluorescence staining and immunoblot analysis. No increase in Ki‐67 expression was observed following staurosporine treatment (Figure S1A,B), indicating that the preserved viability of melanocytes was not associated with enhanced proliferation. These findings further support that the differential response of melanocytes to staurosporine is not attributable to enhanced proliferation but rather reflects resistance to apoptosis.

### 3.2 Staurosporine Induces Dendrite Extension and Melanocyte Maturation‐Associated Phenotypes

**FIGURE 2:**
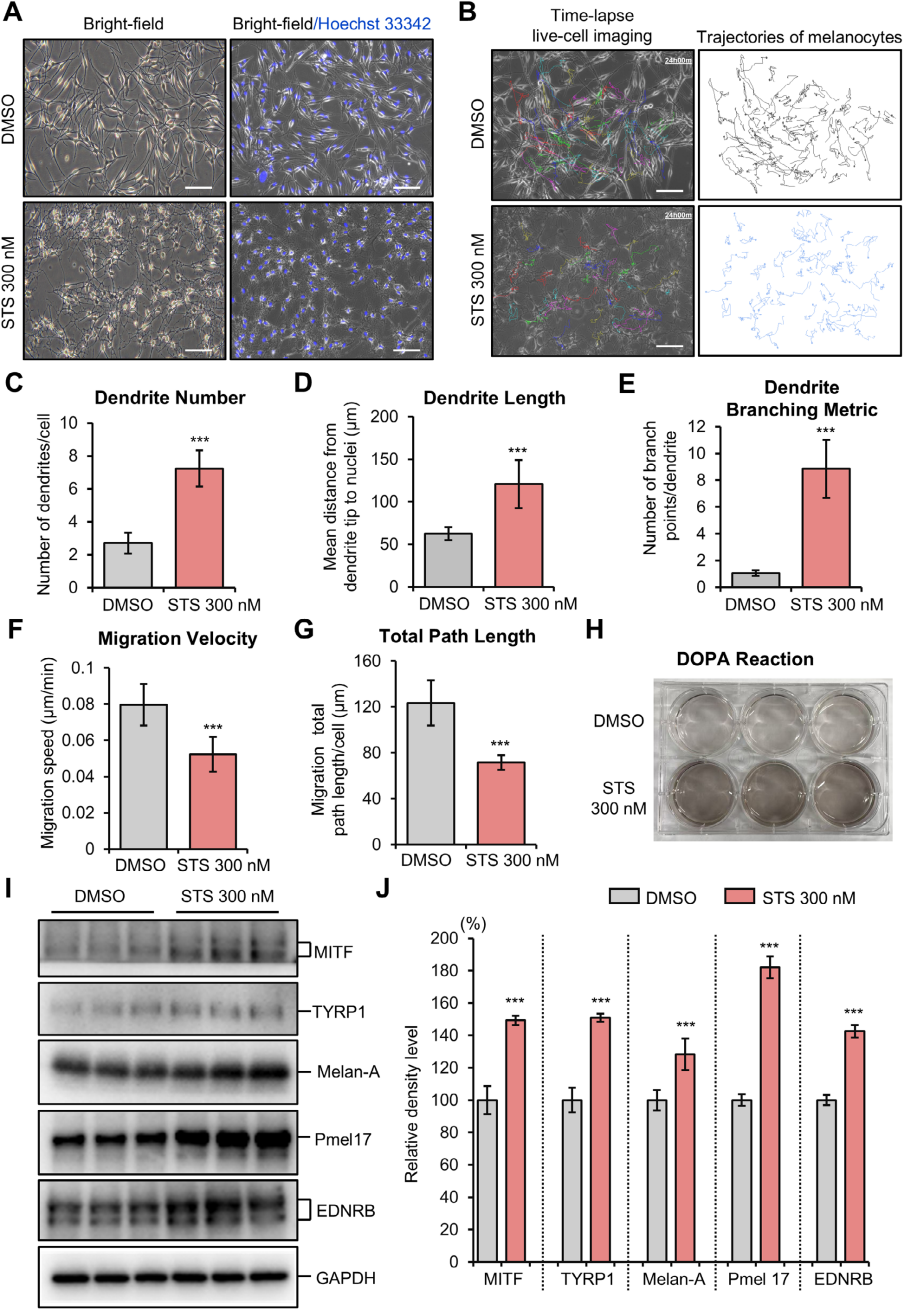
Effects of staurosporine on melanocyte morphology, dendrite formation, migration, and maturation. (A) Representative bright‐field and merged bright‐field/Hoechst 33342 images of HEMn‐MP melanocytes treated with DMSO or 300 nM STS for 24 h. Scale bars, 100 μm. (B) Time‐lapse live‐cell imaging of melanocytes treated with DMSO or STS. Images were acquired every 15 min for 24 h and analyzed by single‐cell tracking. Representative cell trajectories are shown in the right panels. Scale bar, 50 μm. (C–E) Quantification of dendrite number per cell (C), dendrite length (D), and dendrite branching (number of branch points per dendrite) (E) following DMSO or STS treatment. (F, G) Quantification of migration velocity (F) and total migration path length (G) obtained from single‐cell tracking analysis. (H) Representative images of the DOPA reaction in melanocytes treated with DMSO or STS for 72 h. (I) Immunoblot analysis of melanocyte maturation‐ and melanogenesis‐associated proteins (MITF, TYRP1, Melan‐A, PMEL17, and EDNRB) in melanocytes treated with DMSO or STS for 48 h. GAPDH served as the loading control. (J) Quantification of the relative protein expression shown in (I). Protein levels were normalized to GAPDH and expressed relative to the DMSO‐treated control. Data in (C–G) and (J) represent three independent experiments and are presented as the mean ± SD. Statistical significance was determined by an unpaired two‐tailed Student's *t*‐test. ***p < 0.01.

After establishing that staurosporine does not induce apoptosis in normal melanocytes, we observed pronounced morphological changes in melanocytes. Bright‐field imaging and nuclear staining showed that staurosporine‐treated melanocytes remained viable and adherent but underwent marked morphological remodeling compared with dimethyl sulfoxide (DMSO)‐treated controls (Figure 2A and Videos S1 and S2). Specifically, staurosporine‐treated melanocytes exhibited elongated cell bodies and increased dendritic extensions, consistent with enhanced dendrite formation rather than cytotoxic stress. To characterize the dynamic behavior underlying these morphological changes, we performed time‐lapse live‐cell imaging and single‐cell trajectory analysis. Under control conditions, melanocytes exhibited active migration with extended trajectories, whereas staurosporine‐treated melanocytes showed more confined movement with shorter trajectories (Figure 2B and Videos S3 and S4). Quantitative analysis demonstrated that staurosporine significantly increased the number of dendrites per cell (Figure 2C), the mean dendrite length (Figure 2D), and dendrite branching (Figure 2E). In contrast, both migration velocity (Figure 2F) and total migration path length (Figure 2G) were significantly reduced, indicating that staurosporine promotes a transition from a motile phenotype to a more stationary, highly dendritic phenotype characteristic of matured melanocytes.

We next assessed whether these morphological changes were accompanied by enhanced melanogenic activity. 3,4‐Dihydroxyphenylalanine (DOPA) reaction assays demonstrated increased DOPA oxidase activity in staurosporine‐treated melanocytes compared with DMSO‐treated controls (Figure 2H), indicating enhanced melanogenic activity. Consistent with this finding, immunoblot analysis revealed increased expression of melanocyte maturation‐ and melanogenesis‐associated proteins, including microphthalmia‐associated transcription factor (MITF), tyrosinase‐related protein 1 (TYRP1), melanocyte antigen (Melan‐A), premelanosome protein (PMEL17), and endothelin receptor type B (EDNRB), following staurosporine treatment (Figure 2I). Densitometric analysis confirmed significant upregulation of these proteins compared with DMSO‐treated cells (Figure 2J). Collectively, these findings demonstrate that staurosporine induces coordinated functional remodeling of melanocytes, characterized by enhanced dendrite formation, dendrite elongation, increased dendrite branching, reduced migratory behavior, and enhanced melanogenic differentiation. These changes occur in the absence of apoptosis, supporting the conclusion that staurosporine promotes melanocyte maturation rather than cytotoxicity.

### 3.3 Staurosporine Suppresses PKCμ Signaling and Stabilizes β‐Catenin in Melanocytes

**FIGURE 3:**
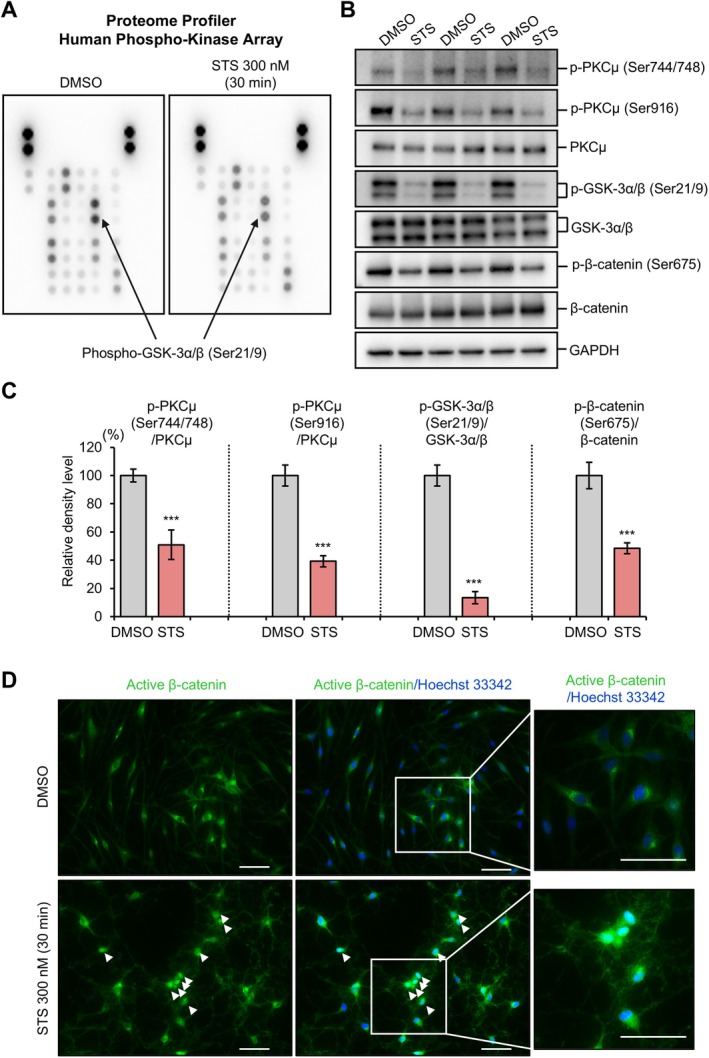
Phospho‐kinase profiling and analysis of staurosporine‐induced signaling pathways in melanocytes. (A) Human phospho‐kinase array analysis of melanocytes treated with DMSO or staurosporine (STS; 300 nM) for 30 min. Representative array images and quantification of selected phosphorylated kinases are shown. (B) Immunoblot analysis of PKD/PKCμ phosphorylation (Ser744/748 and Ser916), total PKD/PKCμ, GSK3α/β phosphorylation (Ser21/9), total GSK3α/β, β‐catenin phosphorylation (Ser675), and total β‐catenin in melanocytes treated with DMSO or STS for 30 min. (C) Quantification of relative protein band intensities shown in (B), normalized to corresponding total protein levels or GAPDH, as indicated. (D) Immunofluorescence staining for active β‐catenin in melanocytes treated with DMSO or STS. Nuclei were counterstained with Hoechst 33342. Representative active β‐catenin‐positive cells are indicated by white arrowheads and shown at higher magnification in right panel. Scale bars, 100 μm. Data in (C) represent three independent experiments and are presented as mean ± SD. Statistical significance was determined by an unpaired two‐tailed Student's *t*‐test. ***p < 0.01.

To clarify signaling mechanisms underlying the staurosporine‐induced melanocyte phenotypic shift, we first performed an unbiased phospho‐kinase profiling analysis. Human phospho‐kinase arrays revealed considerable alterations in kinase phosphorylation after staurosporine treatment, including a pronounced reduction in glycogen synthase kinase 3α/β (GSK3α/β) phosphorylation at Ser21/9 compared with DMSO‐treated controls (Figure 3A), suggesting modulation of β‐catenin regulatory pathways. Staurosporine has long been characterized as a potent, broad‐spectrum PKC inhibitor (Yamamoto et al. 1992); however, PKC isoforms targeted by staurosporine in normal human melanocytes have not been defined. Therefore, we systematically examined the phosphorylation status of major PKC isoforms expressed in melanocytes. Immunoblot analysis showed that staurosporine treatment significantly reduced PKCμ phosphorylation at Ser744/748 and Ser916, consistent with suppression of PKC signaling activity (Figure 3B,C). In contrast, phosphorylation of other PKC isoforms, including PKCα, PKCβII, PKCζ/λ, PKCθ, and PKCδ, remained largely unchanged under the same conditions (Figure S2), indicating that staurosporine has a preferential effect on PKCμ signaling in melanocytes. In parallel, GSK3α/β phosphorylation at Ser21/9 was greatly decreased, suggesting altered regulation of the GSK3‐dependent β‐catenin destruction complex. Concomitantly, β‐catenin phosphorylation at Ser675 was significantly reduced (Figure 3B,C). To determine whether suppression of PKC signaling by staurosporine led to accumulation of transcriptionally active β‐catenin, we performed immunofluorescence staining for active β‐catenin. Whereas control melanocytes showed weak and diffuse staining, staurosporine‐treated melanocytes exhibited a pronounced increase in active β‐catenin‐positive cells, with prominent nuclear localization (Figure 3D). Our findings indicate β‐catenin‐dependent signaling activation downstream of PKC inhibition. Taken together, these data demonstrate that staurosporine suppresses PKC signaling in melanocytes, leading to GSK3α/β activity inhibition and active β‐catenin stabilization. This reprogrammed signaling provides a mechanistic basis for the staurosporine‐induced changes in melanocyte morphology and maturation shown in Figure 2.

### 3.4 Staurosporine‐Induced Alterations in Rho Family Protein Expression Promote Melanocyte Dendrite Formation

**FIGURE 4:**
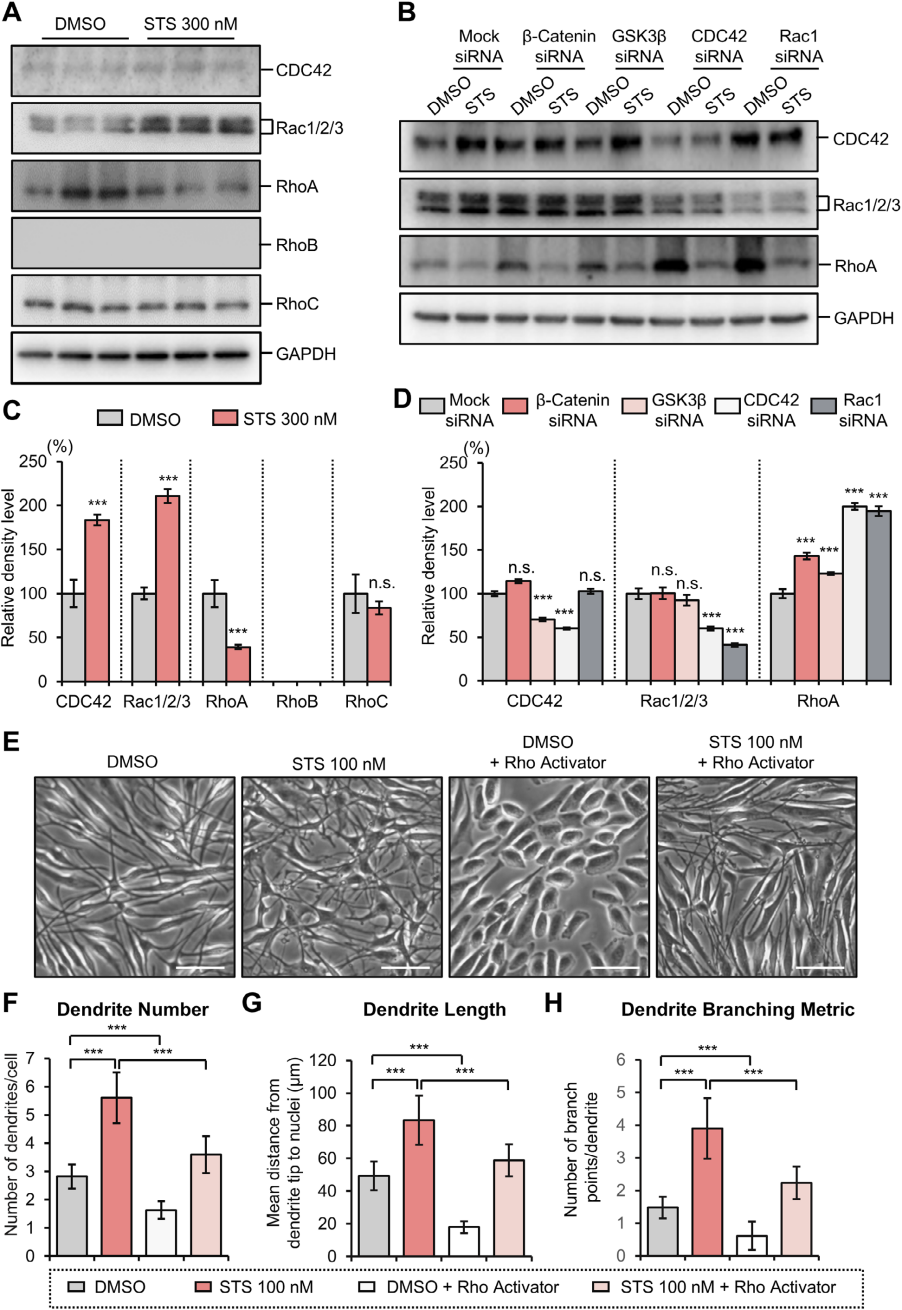
Effects of staurosporine on Rho family protein expression and dendrite remodeling in melanocytes. (A) Immunoblot analysis of CDC42, Rac1/2/3, RhoA, RhoB, and RhoC protein expression in HEMn‐MP melanocytes treated with DMSO or 300 nM STS for 48 h. GAPDH served as the loading control. (B) Immunoblot analysis of CDC42, Rac1/2/3, and RhoA protein expression in melanocytes transfected with mock control siRNA or siRNAs targeting β‐catenin, GSK3β, CDC42, or Rac1 for 24 h, followed by treatment with DMSO or 300 nM STS for an additional 24 h. GAPDH served as the loading control. (C) Densitometric quantification of the protein expression shown in (A). Protein levels were normalized to GAPDH and expressed relative to the DMSO‐treated control. (D) Densitometric quantification of the protein expression shown in (B). Protein levels were normalized to GAPDH and expressed relative to the mock siRNA group. (E) Representative bright‐field images of melanocytes treated with DMSO or 100 nM STS in the absence or presence of a Rho activator (5 μg/mL) for 24 h. Scale bars, 50 μm. (F–H) Quantification of dendrite number per cell (F), mean distance from the dendrite tip to the nucleus (G), and dendrite branching (number of branch points per dendrite) (H) under the conditions shown in (E). Data in (C, D), and (F–H) represent three independent experiments and are presented as the mean ± SD. Statistical significance was determined by an unpaired two‐tailed Student's *t*‐test (C) or one‐way ANOVA followed by Bonferroni's multiple comparisons test (D, F–H). ***p < 0.01; n.s., not significant.

To investigate the molecular basis of staurosporine‐induced morphological changes, we focused on the Rho family of small GTPases, which are central regulators of actin cytoskeleton dynamics and dendrite formation in melanocytes (Scott 2002). Immunoblot analysis demonstrated that staurosporine treatment (300 nM) significantly increased protein levels of cell division cycle 42 (CDC42) and Ras‐related C3 botulinum toxin substrate 1/2/3 (Rac1/2/3), while concomitantly reducing RhoA expression (Figure 4A,C). In contrast, expression patterns of Ras homolog family member B (RhoB) and Ras homolog family member C (RhoC) remained largely unchanged, indicating selective modulation of specific Rho GTPase family members.

To define upstream regulatory relationships governing RhoA expression, we transfected melanocytes with small interfering RNAs (siRNAs) targeting β‐catenin, GSK‐3β, CDC42, or Rac1. Knockdown of β‐catenin or GSK‐3β resulted in a pronounced increase in RhoA protein levels compared with control siRNA, indicating that GSK‐3β and β‐catenin signaling function upstream to restrain RhoA expression (Figure 4B,D). Notably, depletion of CDC42 or Rac1 led to pronounced upregulation of RhoA, supporting a model in which both CDC42 and Rac1 act as upstream suppressors of RhoA expression in melanocytes. Consistent with this regulatory hierarchy, pharmacological activation of RhoA markedly attenuated staurosporine‐induced dendrite formation, resulting in significant reductions in dendrite number, dendrite length, and dendrite branching (Figure 4E–H), supporting a central role for RhoA suppression in dendrite formation. Collectively, these findings indicate that coordinated suppression of RhoA expression by upstream β‐catenin, CDC42, and Rac1 is a key molecular feature associated with enhanced melanocyte dendrite remodeling.

### 3.5 Topical Staurosporine Enhances Epidermal Pigmentation and Promotes Melanocyte Maturation in Guinea Pig Skin

**FIGURE 5:**
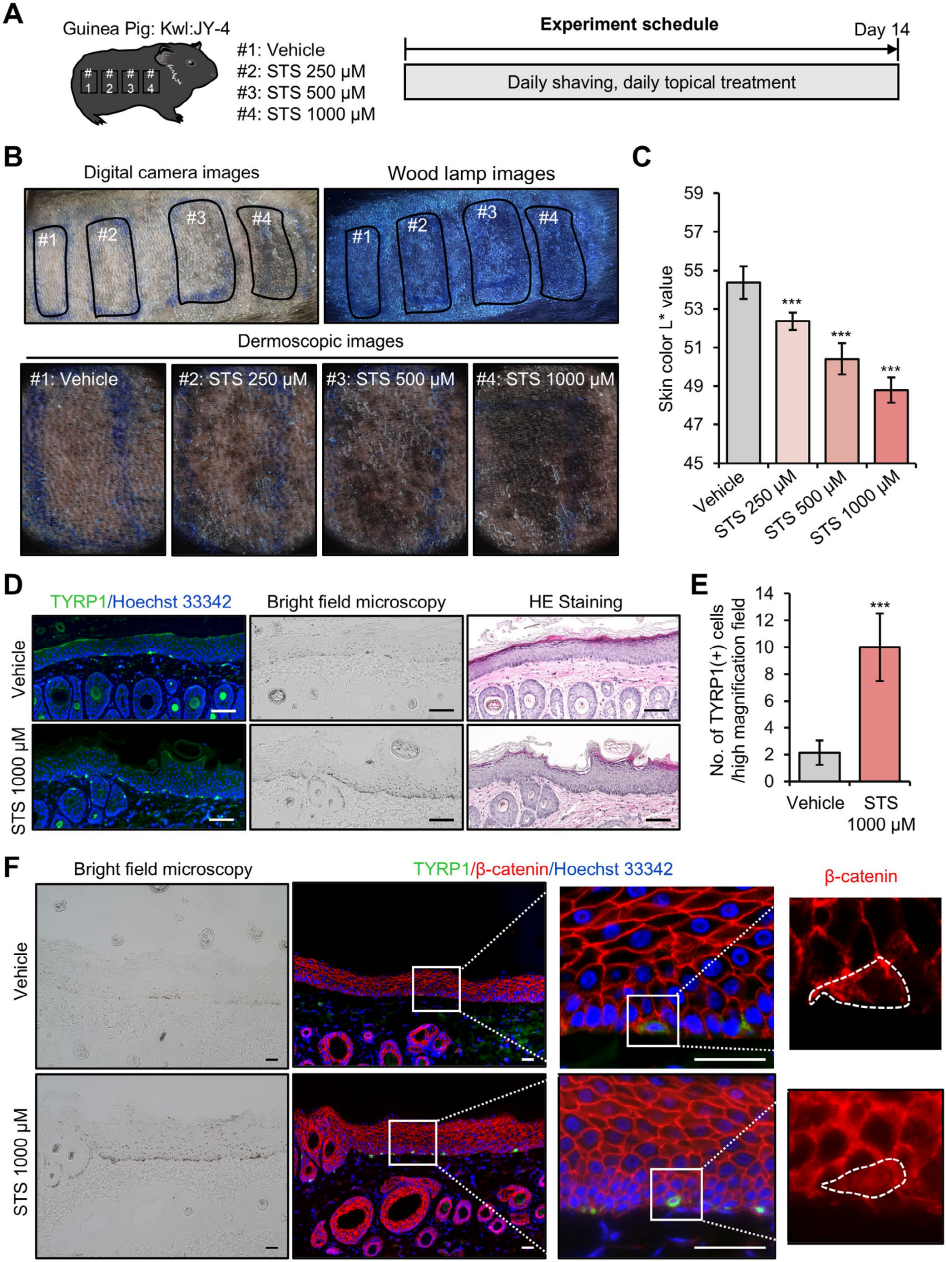
Topical staurosporine promotes skin pigmentation and epidermal melanocyte accumulation in guinea pigs. (A) Experimental schedule. (B) Representative digital photographs, Wood lamp images, and dermoscopic images of treated skin areas after 14 days of topical application. (C) Quantification of skin color L* values for the treated areas shown in (B). (D) Representative images of TYRP1 immunofluorescence (green) with Hoechst 33342 nuclear counterstaining (blue), bright‐field microscopy, and hematoxylin and eosin staining of skin sections from vehicle‐ and STS‐treated areas (1000 μM). White scale bar, 100 μm. (E) Quantification of TYRP1‐positive cells per high‐power field. (F) Representative bright‐field images and immunofluorescence staining for TYRP1 (green), β‐catenin (red), and nuclei (Hoechst 33342; blue) in skin sections from vehicle‐ and STS‐treated sites (1000 μM). Enlarged views highlight β‐catenin localization in epidermal melanocytes (outlined by dashed lines). Scale bar, 50 μm. Data in (C) and (E) are presented as mean ± SD. Statistical significance in (C) was determined by one‐way ANOVA followed by Bonferroni's multiple comparisons test, whereas statistical significance in (E) was determined by an unpaired two‐tailed Student's *t*‐test. ***p < 0.01.

To determine whether the pro‐maturation and dendrite‐promoting effects observed in vitro translate to an in vivo setting, we assessed the effects of topical staurosporine on guinea pig skin pigmentation. Dorsal skin areas were shaved and treated daily with vehicle or increasing concentrations of staurosporine for 14 days (Figure 5A). Macroscopic examination revealed dose‐dependent darkening of treated skin after topical staurosporine application, as evidenced by digital photography, wood lamp examination, and dermoscopic analysis (Figure 5B). Quantitative colorimetric assessment confirmed a significant, dose‐dependent decrease in skin color L* values in staurosporine‐treated sites compared with vehicle controls, indicating enhanced pigmentation (Figure 5C). Histological and immunofluorescence analyses further supported these findings. TYRP1 immunostaining demonstrated a substantial increase in the number of TYRP1‐positive melanocytes along the basal epidermis in staurosporine‐treated skin compared with vehicle‐treated controls (Figure 5D,E). Hematoxylin and eosin staining showed preserved epidermal architecture without overt tissue damage or inflammation, indicating good local tolerability of topical staurosporine treatment (Figure 5D). To determine whether the increased number of TYRP1‐positive melanocytes was associated with enhanced proliferation, Ki‐67 immunofluorescence staining was performed. Ki‐67‐positive epidermal melanocytes were rarely detected under either vehicle‐ or staurosporine‐treated conditions, whereas basal keratinocytes showed comparable Ki‐67 positivity in both groups (Figure S3A,B). In contrast, TYRP1 fluorescence intensity per epidermal melanocyte was significantly increased following staurosporine treatment (Figure S3C), indicating enhanced melanocyte maturation rather than increased proliferative activity. Consistent with the in vitro findings, staurosporine‐treated skin exhibited a pronounced increase in nuclear β‐catenin localization within epidermal melanocytes, accompanied by enhanced TYRP1 expression (Figure 5F). These findings indicate that topical staurosporine enhances melanocyte maturation and increases the abundance of TYRP1‐positive melanocytes within the epidermis in association with β‐catenin signaling, without inducing melanocyte proliferation. Collectively, our observations demonstrate that topical staurosporine enhances epidermal pigmentation and promotes melanocyte maturation in vivo, supporting its translational potential as a topical modulator of melanocyte function.

### 3.6 Topical Staurosporine Promotes Repigmentation and Epidermal Melanocyte Recovery in RD‐Induced Leukoderma

**FIGURE 6:**
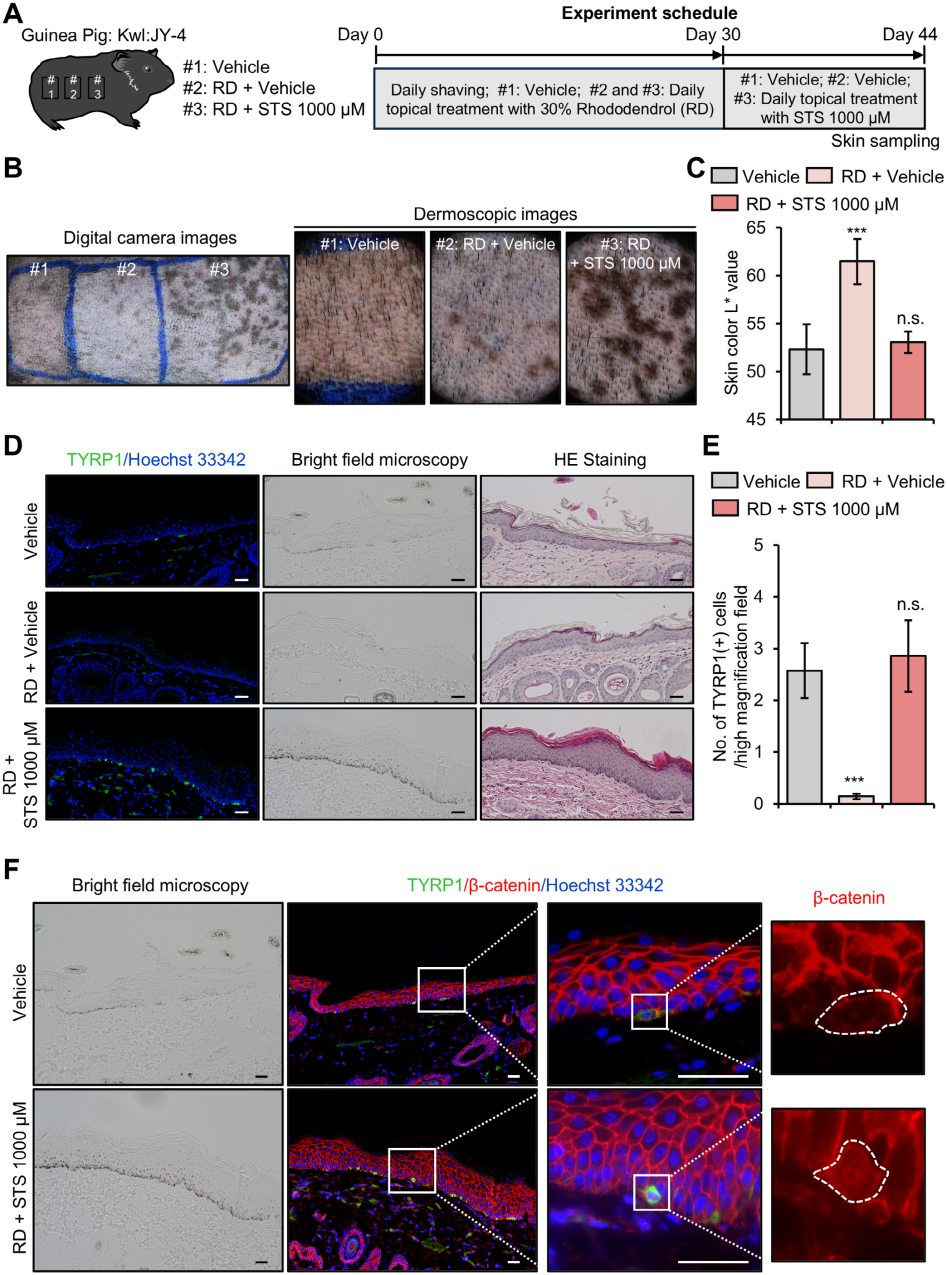
Topical staurosporine enhances repigmentation in guinea pigs with rhododendrol‐induced depigmentation. (A) Experimental schedule. (B) Representative digital photographs and dermoscopic images of skin areas under the indicated treatments. (C) Quantification of skin color L* values in the indicated treatment groups. (D) Representative immunofluorescence staining for TYRP1 (green) with Hoechst 33342 nuclear counterstaining (blue), together with bright‐field microscopy and hematoxylin and eosin staining of skin sections from each group. Scale bar, 50 μm. (E) Quantification of TYRP1‐positive cells per high‐power field. (F) Representative bright‐field and immunofluorescence images showing TYRP1 (green), β‐catenin (red), and nuclear counterstaining (Hoechst 33342; blue). Enlarged views highlight epidermal melanocytes and β‐catenin localization. Data in (C, E) are presented as mean ± SD. Statistical significance in (C) was determined by one‐way ANOVA followed by Bonferroni's multiple comparisons test, whereas statistical significance in (E) was determined by an unpaired two‐tailed Student's *t*‐test. ***p < 0.01.

To assess the therapeutic effects of topical staurosporine in depigmentation disorders, we used an RD‐induced leukoderma guinea pig model, which recapitulates chemical‐induced melanocyte loss and depigmentation in vivo (Inoue et al. 2021; Kuroda et al. 2014). Repeated topical application of 30% RD for 30 days resulted in obvious skin depigmentation (Figure 6A,B). After cessation of RD treatment, slight spontaneous recovery of skin pigmentation and epidermal melanocytes was observed over the subsequent 14 days. In contrast, topical application of staurosporine (1000 μM) markedly enhanced repigmentation in RD‐treated skin and significantly attenuated RD‐induced skin lightening compared with vehicle‐treated RD skin (Figure 6B,C). Immunofluorescence analysis demonstrated a marked recovery of TYRP1‐positive epidermal melanocytes following topical staurosporine treatment, whereas vehicle‐treated RD skin contained only a few residual melanocytes (Figure 6D,E). Hematoxylin and eosin staining showed no apparent epidermal damage or inflammatory changes following topical staurosporine treatment (Figure 6D). To determine whether melanocyte recovery was associated with increased proliferation, Ki‐67 immunofluorescence staining was performed. Similar to the findings in normally pigmented skin, Ki‐67‐positive epidermal melanocytes were rarely detected in either vehicle‐treated controls or staurosporine‐treated RD skin, whereas the percentage of Ki‐67‐positive basal keratinocytes remained comparable between groups (Figure S4A,B). In contrast, TYRP1 fluorescence intensity per epidermal melanocyte was significantly increased following topical staurosporine treatment (Figure S4C), indicating enhanced melanocyte maturation rather than increased proliferative activity. Finally, epidermal melanocytes in staurosporine‐treated skin exhibited increased nuclear localization of β‐catenin together with enhanced TYRP1 expression compared with vehicle‐treated controls (Figure 6F). These findings indicate that topical staurosporine promotes repigmentation through melanocyte recovery and maturation, without inducing melanocyte proliferation.

## 4 Discussion

Staurosporine has long been recognized as a potent, broad‐spectrum kinase inhibitor and a canonical inducer of apoptosis in melanoma and other malignant cells (Gescher 1998; Omura et al. 1995; Yamamoto et al. 1992). Beyond its cytotoxic effects, accumulating evidence from non‐melanocytic systems indicates that, at nanomolar and sub‐cytotoxic concentrations, staurosporine can promote cellular differentiation and profound morphological remodeling (Borge et al. 1997; Kronfeld et al. 1995; Kulyk 1991; Rasouly et al. 1996; Sano et al. 1994; Thompson and Levin 2010). Such effects have been particularly well documented in neuronal and mesenchymal lineages, where staurosporine induces neurite outgrowth, cytoskeletal reorganization, and lineage‐specific maturation. Despite these observations, the impact of staurosporine on normal human melanocyte biology has remained largely unexplored.

In this study, we demonstrate that staurosporine elicits fundamentally distinct, cell type‐specific responses in normal melanocytes compared with melanoma cells. While melanoma cells undergo apoptosis in response to staurosporine, primary human melanocytes display marked resistance to cell death without evidence of increased proliferative activity and instead activate a robust maturation program. This maturation response occurred without detectable induction of Ki‐67 expression both in vitro and in vivo, further supporting a maturation‐driven rather than proliferation‐driven mechanism. At sub‐cytotoxic concentrations, staurosporine promotes dendritic elongation, enhances melanogenic activity, and increases functional pigmentation in vitro and in vivo. These findings highlight the intrinsic capacity of melanocytes to interpret kinase inhibition signals in a maturation‐promoting rather than cytotoxic manner.

Our data suggests that inhibition of PKC‐dependent signaling relieves constraints on melanocyte maturation, enabling coordinated regulation of pigmentation and cell morphology. This response is associated with stabilization and nuclear accumulation of β‐catenin, a key regulator of melanocyte maturation and lineage maintenance (Gallagher et al. 2013; Larue et al. 2003; Yamada et al. 2013), as well as with coordinated remodeling of Rho family protein expression, which regulates actin cytoskeletal dynamics (Lacour et al. 1992; Scott 2002; Scott and Cassidy 1998). Although β‐catenin is classically activated through canonical Wnt signaling (Yamada et al. 2013), our findings indicate that, in melanocytes, β‐catenin‐dependent differentiation can be engaged through a non‐canonical, Wnt‐independent mechanism. This distinction underscores the context‐dependent roles of β‐catenin signaling in pigment cells, where it favors terminal differentiation and reduced motility rather than proliferation.

Importantly, the coordinated enhancement of dendrite formation, elongation, and branching, together with activation of melanogenic gene expression, provides both the structural and functional basis for efficient pigment production and melanosome transfer. By simultaneously enhancing the biosynthetic capacity of melanocytes and the dendritic network required for pigment delivery, staurosporine engages a comprehensive maturation program that mirrors key features of physiological pigmentation.

The physiological relevance of this response was supported by in vivo experiments demonstrating increased pigmentation following topical staurosporine application in guinea pig skin without overt inflammation or tissue damage. Notably, Ki‐67 staining remained essentially absent in epidermal melanocytes under both normal and depigmented conditions, whereas TYRP1 expression per melanocyte was significantly increased. These findings indicate that the increased number of TYRP1‐positive melanocytes should not be interpreted as evidence of enhanced melanocyte proliferation but rather reflect functional maturation of existing melanocytes. Moreover, staurosporine accelerated repigmentation in a rhododendrol‐induced leukoderma model, characterized by restoration of epidermal melanocytes and increased nuclear β‐catenin localization. Together, these observations indicate that activation of melanocyte‐intrinsic maturation pathways is sufficient to enhance pigmentation under both normal and depigmented conditions.

Epidermal melanocytes are generally regarded as long‐lived, low‐proliferative cells that maintain skin pigmentation primarily through functional plasticity rather than continuous cell turnover (Cichorek et al. 2013; Kondo and Hearing 2011; Yamaguchi et al. 2007). Under physiological conditions, mature melanocytes rarely undergo active proliferation, and dynamic changes in skin pigmentation are therefore thought to rely predominantly on modulation of the functional properties of existing melanocytes rather than expansion of the melanocyte population. Consistent with these intrinsic biological characteristics, we observed no induction of Ki‐67 expression in either cultured melanocytes or guinea pig skin following staurosporine treatment, indicating that the enhanced pigmentation induced by staurosporine is not attributable to increased melanocyte proliferation.

Recent studies have demonstrated that epidermal melanocytes are not a homogeneous population but instead comprise multiple subpopulations representing distinct functional and maturation states (Belote et al. 2021; Brombin and Patton 2024; Kondo and Hearing 2011; Mort et al. 2015; Vandamme and Berx 2019; Yamaguchi et al. 2007; Yang et al. 2026). This cellular heterogeneity provides melanocytes with the capacity to dynamically adjust pigmentation, dendritic morphology, and melanogenic gene expression in response to physiological or pharmacological stimuli. From this perspective, the increased TYRP1 expression together with the absence of Ki‐67 induction observed in the present study suggests that staurosporine promotes the functional maturation of existing melanocytes rather than expanding the melanocyte population through proliferation. This interpretation is further supported by the concomitant activation of β‐catenin signaling, which has been implicated in maintaining melanocyte identity and promoting melanocyte maturation.

The differential sensitivity of normal melanocytes and melanoma cells to staurosporine‐induced apoptosis highlights fundamental differences in kinase network regulation between benign and malignant pigment cells. Whereas oncogenic rewiring in melanoma predisposes cells to apoptosis upon kinase inhibition, normal melanocytes appear capable of maintaining cellular homeostasis and redirecting these signals toward melanocyte maturation. In contrast to ultraviolet radiation‐dependent pigmentation, which relies heavily on keratinocyte‐derived paracrine factors (Gordon et al. 1989; Halaban et al. 1988; Tomita et al. 1987; Yaar et al. 1991), the pathway identified here represents an intrinsic, ultraviolet‐independent mechanism that directly links cytoskeletal remodeling with melanocyte maturation.

Several limitations should be acknowledged. Although our findings support a predominantly melanocyte‐intrinsic mechanism, potential contributions from the epidermal microenvironment, including keratinocyte‐derived signals and neurocutaneous interactions (Hara et al. 1996; Kippenberger et al. 1998), cannot be excluded. In addition, although our data indicate that staurosporine enhances pigmentation without inducing melanocyte proliferation, the present study did not directly trace melanocyte lineage or fate. Therefore, we cannot definitively distinguish whether the increased number of TYRP1‐positive melanocytes in vivo reflects functional maturation of residual melanocytes, de novo melanocyte repopulation, or a combination of both mechanisms. Future lineage‐tracing studies will be required to address this question. Nevertheless, the absence of Ki‐67 induction together with the increased TYRP1 expression per melanocyte strongly supports functional maturation of existing melanocytes as a major contributor to the observed repigmentation.

In summary, our study identifies staurosporine as a potent modulator of melanocyte maturation, dendrite remodeling, and pigmentation through a non‐canonical β‐catenin‐dependent mechanism. Importantly, these effects occur without inducing melanocyte proliferation, highlighting staurosporine as a potential strategy for enhancing pigmentation by promoting functional melanocyte maturation rather than proliferative expansion.

## Author Contributions

**Sylvia Lai:** methodology, data curation, formal analysis, investigation. **Kangning Xu:** data curation, investigation, validation, formal analysis, writing – original draft. **Ichiro Katayama:** supervision, writing – review and editing, project administration. **Fei Yang:** methodology, software, data curation, formal analysis. **Daisuke Tsuruta:** writing – review and editing, supervision. **Yasutaka Kuroda:** validation, data curation. **Lingli Yang:** conceptualization, methodology, software, supervision, validation, visualization, project administration, writing – review and editing.

## Conflicts of Interest

The authors declare no conflicts of interest.

## Supporting information


**Figure S1:** Ki‐67 expression in HEMn‐MP melanocytes following staurosporine treatment in vitro. (A) Representative immunofluorescence images of Ki‐67 (green) and nuclei stained with Hoechst 33342 (blue) in HEMn‐MP melanocytes treated with DMSO or 300 nM staurosporine (STS) for 48 h. Immortalized human keratinocytes (PSVK1) were included as a positive control for Ki‐67 expression. Bright‐field and merged images are shown. Scale bars, 100 μm. (B) Representative immunoblot analysis of Ki‐67 protein expression in HEMn‐MP melanocytes treated with DMSO or 300 nM STS for 48 h. PSVK1 cells, normal human dermal fibroblasts (NHDF), and human macrophages (M0, M1, and M2) were included for comparison. GAPDH served as the loading control.
**Figure S2:** Effects of staurosporine on PKC isoform phosphorylation in cultured human epidermal melanocytes. Representative Western blot analysis showing the phosphorylation status and total protein levels of the indicated PKC isoforms in melanocytes treated with 300 nM staurosporine for the indicated time periods. Total protein levels of PKCμ, PKCα, PKCζ, and PKCδ are shown as indicated. GAPDH served as the loading control.
**Figure S3:** Ki‐67 and TYRP1 expression in normal guinea pig epidermal melanocytes following topical staurosporine treatment in vivo. (A) Representative bright‐field and immunofluorescence images of normal guinea pig skin treated topically with vehicle or 1000 μM STS for 14 days. TYRP1 is shown in green, Ki‐67 in red, and nuclei were counterstained with Hoechst 33342 (blue). Enlarged views of the boxed regions and the corresponding Ki‐67 channel are shown on the right. Scale bars, 50 μm. (B) Quantification of the percentage of Ki‐67‐positive cells in basal keratinocytes (Basal KC) and epidermal melanocytes (Epidermal MC) in vehicle‐ and STS‐treated skin. (C) Quantification of the relative TYRP1 expression per epidermal melanocyte in vehicle‐ and STS‐treated skin. Data are presented as the mean ± SD. Statistical significance was determined by an unpaired two‐tailed Student's *t*‐test. ***p < 0.01.
**Figure S4:** Ki‐67 and TYRP1 expression in epidermal melanocytes in the rhododendrol‐induced depigmentation model following topical staurosporine treatment in vivo. (A) Representative bright‐field and immunofluorescence images of normal guinea pig skin (Vehicle) and rhododendrol (RD)‐treated skin after topical application of 1000 μM STS for 14 days following 30 days of RD treatment. TYRP1 is shown in green, Ki‐67 in red, and nuclei were counterstained with Hoechst 33342 (blue). Enlarged views of the boxed regions and the corresponding Ki‐67 channel are shown on the right. Scale bars, 50 μm. (B) Quantification of the percentage of Ki‐67‐positive basal keratinocytes (Basal KC) and epidermal melanocytes (Epidermal MC) in vehicle‐ and STS‐treated skin. (C) Quantification of the relative TYRP1 expression per epidermal melanocyte in vehicle‐ and STS‐treated skin. Data are presented as the mean ± SD. Statistical significance was determined by an unpaired two‐tailed Student's *t*‐test. ***p < 0.01.

**Video S1:** Time‐lapse live‐cell imaging of cultured HEMn‐MPs with dimethyl sulfoxide (DMSO) treatment as the control.

**Video S2:** Time‐lapse live‐cell imaging of cultured HEMn‐MPs with 300 nM staurosporine treatment.

**Video S3:** Time‐lapse imaging of cultured HEMn‐MPs with dimethyl sulfoxide (DMSO) treatment as the control. A single‐cell tracking assay was employed to monitor the movement of melanocytes.

**Video S4:** Time‐lapse imaging of cultured HEMn‐MPs with 300 nM staurosporine treatment. A single‐cell tracking assay was employed to monitor the movement of melanocytes.

## Data Availability

The data that support the findings of this study are available from the corresponding author upon reasonable request.
