# Supplementary material for: Staurosporine Drives Non‐Canonical Melanocyte Maturation by Coupling β‐Catenin Signaling to Actin‐Dependent Dendrite Remodeling
Source: Pigment Cell Melanoma Res. 2026 Aug 13;39(5):e70116. doi: 10.1111/pcmr.70116 (PMC13473815; doi:10.1111/pcmr.70116)
Supplement: Supplementary file 1 [file PCMR-39-0-s003.docx]

**Supplementary information**

**Supplementary materials & methods**

1. *Cell lines and cell culture*

PSVK1 cells (immortalized human keratinocytes) and NHDF cells (normal human dermal fibroblasts) were cultured, and human macrophages (M0, M1, and M2) were prepared, as previously described (Zhu et al., 2026). PSVK1 cells served as a positive control for Ki67 expression, whereas NHDF cells and macrophages (M0, M1, and M2) were included for comparison of basal Ki67 expression among different human skin-related cell types.

2. *Western blot analysis*

A 5-µg aliquot of the extracted proteins was utilized for western blot analysis. Primary antibodies were employed at the following dilutions: anti-Ki-67 (CST, Danvers, MA, USA) at 1:500, anti-p-PKCμ (Ser916) (CST) at 1:1000, anti-p-PKCμ (Ser744/748) (CST) at 1:1000, anti-PKCμ (CST) at 1:1000, anti-p-PKCα/βII (Thr638/641) (CST) at 1:1000, anti-PKCα (CST) at 1:1000, anti-p-PKCζ/λ (Thr410/403) (CST) at 1:1000, anti-PKCζ (CST) at 1:1000, anti-p-PKCδ (Thr505) (CST) at 1:1000, anti-p-PKCδ/θ (Ser643/676) (CST) at 1:1000, anti-p-PKCθ (Thr538) (CST) at 1:1000, anti-PKCδ (CST) at 1:1000, and anti-GAPDH (CST) at 1:1000. The anti-GAPDH antibody was employed as a loading control.

3. *Immunofluorescence staining of cultured cells*

HEMn-MPs were treated with DMSO or 300 nM staurosporine (STS) for 48 hours, whereas PSVK1 cells were included as a positive control for Ki-67 immunofluorescence staining. Cells were fixed with 4% paraformaldehyde for 5 min and incubated overnight at 4°C with an anti-Ki-67 antibody (1:100; Dako, Santa Clara, CA, USA). After washing, cells were incubated with Alexa Fluor-conjugated secondary antibodies (Invitrogen, Waltham, MA, USA). Nuclei were counterstained with Hoechst 33342 (1:500; Invitrogen), and bright-field and fluorescence images were acquired using a Biozero BZ-8100 fluorescence microscope (Keyence Corporation, Osaka, Japan).

4. *Immunofluorescence staining of guinea pig skin sections*

Dorsal skin tissues were fixed in 10% neutral-buffered formalin, embedded in paraffin, and sectioned at a thickness of 5 μm. Following deparaffinization and antigen retrieval, the sections were incubated overnight at 4°C with primary antibodies against tyrosinase-related protein 1 (TYRP1; 1:200; Sigma-Aldrich, St. Louis, MO, USA) and Ki-67 (1:100; DAKO). The sections were then incubated with Alexa Fluor 488-conjugated goat anti-rabbit IgG and Alexa Fluor 555-conjugated goat anti-mouse IgG secondary antibodies (Invitrogen). Nuclei were counterstained with Hoechst 33342 (1:500; Invitrogen). Fluorescence images were captured using a Biozero BZ-8100 confocal microscope (Keyence Corporation). For quantitative analysis, Ki-67-positive melanocytes were identified as TYRP1-positive cells showing nuclear Ki-67 immunoreactivity. The percentage of Ki-67-wereitive melanocytes was calculated by dividing the number of Ki-67-positive TYRP1-positive cells by the total number of TYRP1-positive melanocytes. The percentage of Ki-67-positive basal keratinocytes was calculated by dividing the number of Ki-67-positive basal keratinocytes by the total number of basal keratinocytes. TYRP1 fluorescence intensity was quantified using ImageJ software (National Institutes of Health, Bethesda, MD, USA) and normalized to the number of TYRP1-positive melanocytes to obtain the relative TYRP1 expression per epidermal melanocyte.

**Supplementary figure legends**

**Supplementary Figure S1. Ki-67 expression in HEMn-MP melanocytes following staurosporine treatment *in vitro*.** **(A)** Representative immunofluorescence images of Ki-67 (green) and nuclei stained with Hoechst 33342 (blue) in HEMn-MP melanocytes treated with DMSO or 300 nM staurosporine (STS) for 48 hours. Immortalized human keratinocytes (PSVK1) were included as a positive control for Ki-67 expression. Bright-field and merged images are shown. Scale bars, 100 μm. **(B)** Representative immunoblot analysis of Ki-67 protein expression in HEMn-MP melanocytes treated with DMSO or 300 nM STS for 48 hours. PSVK1 cells, normal human dermal fibroblasts (NHDF), and human macrophages (M0, M1, and M2) were included for comparison. GAPDH served as the loading control.

**Supplementary Figure S2.** **Effects of staurosporine on PKC isoform phosphorylation in cultured human epidermal melanocytes.** Representative Western blot analysis showing the phosphorylation status and total protein levels of the indicated PKC isoforms in melanocytes treated with 300 nM staurosporine for the indicated time periods. Total protein levels of PKCμ, PKCα, PKCζ, and PKCδ are shown as indicated. GAPDH served as the loading control.

**Supplementary Figure S3.** **Ki-67 and TYRP1 expression in normal guinea pig epidermal melanocytes following topical staurosporine treatment *in vivo*.** **(A)** Representative bright-field and immunofluorescence images of normal guinea pig skin treated topically with vehicle or 1000 μM STS for 14 days. TYRP1 is shown in green, Ki-67 in red, and nuclei were counterstained with Hoechst 33342 (blue). Enlarged views of the boxed regions and the corresponding Ki-67 channel are shown on the right. Scale bars, 50 μm. **(B)** Quantification of the percentage of Ki-67-positive cells in basal keratinocytes (Basal KC) and epidermal melanocytes (Epidermal MC) in vehicle- and STS-treated skin. **(C)** Quantification of the relative TYRP1 expression per epidermal melanocyte in vehicle- and STS-treated skin. Data are presented as the mean ± SD. Statistical significance was determined by an unpaired two-tailed Student's *t*-test. ^***^*P* < 0.01.

**Supplementary Figure S4.** **Ki-67 and TYRP1 expression in epidermal melanocytes in the rhododendrol-induced depigmentation model following topical staurosporine treatment *in vivo*.** **(A)** Representative bright-field and immunofluorescence images of normal guinea pig skin (Vehicle) and rhododendrol (RD)-treated skin after topical application of 1000 μM STS for 14 days following 30 days of RD treatment. TYRP1 is shown in green, Ki-67 in red, and nuclei were counterstained with Hoechst 33342 (blue). Enlarged views of the boxed regions and the corresponding Ki-67 channel are shown on the right. Scale bars, 50 μm. **(B)** Quantification of the percentage of Ki-67-positive basal keratinocytes (Basal KC) and epidermal melanocytes (Epidermal MC) in vehicle- and STS-treated skin. **(C)** Quantification of the relative TYRP1 expression per epidermal melanocyte in vehicle- and STS-treated skin. Data are presented as the mean ± SD. Statistical significance was determined by an unpaired two-tailed Student's *t*-test. ^***^*P* < 0.01.

**Supplementary video legends**

**Supplementary Video S1:** Time-lapse live-cell imaging of cultured HEMn-MPs with dimethyl sulfoxide (DMSO) treatment as the control.

**Supplementary Video S2:** Time-lapse live-cell imaging of cultured HEMn-MPs with 300 nM staurosporine treatment.

**Supplementary Video S3:** Time-lapse imaging of cultured HEMn-MPs with dimethyl sulfoxide (DMSO) treatment as the control. A single-cell tracking assay was employed to monitor the movement of melanocytes.

**Supplementary Video S4:** Time-lapse imaging of cultured HEMn-MPs with 300 nM staurosporine treatment. A single-cell tracking assay was employed to monitor the movement of melanocytes.

Zhu, A., Yang, L., Lai, S., Yang, F., Tsuruta, D., & Katayama, I. (2026). Circadian Clock Regulates Epidermal Endocrine System in Homeostatic Skin Pigmentation. *Experimental Dermatology, 35*(6), e70296. doi:10.1111/exd.70296

**Supplementary Figure 1.**

**Supplementary Figure 2.**

**Supplementary Figure 3.**

**Supplementary Figure 4.**
